# Real-Time Human Detection and Gesture Recognition for On-Board UAV Rescue

**DOI:** 10.3390/s21062180

**Published:** 2021-03-20

**Authors:** Chang Liu, Tamás Szirányi

**Affiliations:** 1 Department of Networked Systems and Services, Budapest University of Technology and Economics, BME Informatika épület Magyar tudósok körútja 2, 1117 Budapest, Hungary; 2 Machine Perception Research Laboratory of Institute for Computer Science and Control (SZTAKI), Kende u. 13-17, 1111 Budapest, Hungary

**Keywords:** unmanned aerial vehicles (UAVs), search and rescue (SAR), UAV human communication, body gesture recognition, hand gesture recognition, neural networks, deep learning

## Abstract

Unmanned aerial vehicles (UAVs) play an important role in numerous technical and scientific fields, especially in wilderness rescue. This paper carries out work on real-time UAV human detection and recognition of body and hand rescue gestures. We use body-featuring solutions to establish biometric communications, like yolo3-tiny for human detection. When the presence of a person is detected, the system will enter the gesture recognition phase, where the user and the drone can communicate briefly and effectively, avoiding the drawbacks of speech communication. A data-set of ten body rescue gestures (i.e., Kick, Punch, Squat, Stand, Attention, Cancel, Walk, Sit, Direction, and PhoneCall) has been created by a UAV on-board camera. The two most important gestures are the novel dynamic Attention and Cancel which represent the set and reset functions respectively. When the rescue gesture of the human body is recognized as Attention, the drone will gradually approach the user with a larger resolution for hand gesture recognition. The system achieves 99.80% accuracy on testing data in body gesture data-set and 94.71% accuracy on testing data in hand gesture data-set by using the deep learning method. Experiments conducted on real-time UAV cameras confirm our solution can achieve our expected UAV rescue purpose.

## 1. Introduction

With the development of science and technology, especially computer vision technology, the application of unmanned aerial vehicles (UAVs) in various fields is becoming more and more widespread, such as photogrammetry [1], agriculture [2], forestry [3], remote sensing [4], monitoring [5], and search and rescue [6,7]. Drones are more mobile and versatile, and therefore more efficient, than surveillance cameras with fixed angles, proportions, and views. With these advantages, combined with the state-of-art computer vision technology, drones are therefore finding important applications in a wide range of fields. Increasingly researchers have made numerous significant research outcomes in these two intersecting areas. For example, vision-based methods for UAV navigation [8], UAV-based computer vision for an airboat navigation in paddy field [9], deep learning techniques for estimation of the yield and size of citrus fruits using a UAV [10], drone pedestrian detection [11], hand gesture recognition for UAV control [12]. It is also essential to apply the latest computer vision technology to the field of drone wilderness rescue. The layered search and rescue (LSAR) algorithm was carried out for multi-UAVs search and rescue missions [13]. An Embedded system was implemented with the capability of detecting open water swimmers by deep learning techniques [14]. The detection and monitoring of forest fires have been achieved using unmanned aerial vehicles to reduce the number of false alarms of forest fires [15]. The use of a drone with an on-board voice recognition system to detect victims in earthquakes was realized in [16]. UAV has the ability to overcome the problem of fixed coverage and it also can reach difficult access areas. Therefore, it will provide great help to human beings in need of rescue.

Drone rescue generally takes place in a wilderness environment and there are certain drawbacks to rescue work via speech, as speech recognition [17] is more dependent on the external environment, however, we cannot avoid some of the noise [18] generated by the external environment (e.g., rotor noise), which makes it impossible to carry out rescue work effectively. Another disadvantage of speech communication between drones and humans on the ground in noisy environments is that there are many different possible languages spoken in touristic sites and even the same language can have different meanings in some cases [19], making it impossible for drones to understand the questions posed by humans in some cases. Due to these problems, a limited and well-oriented dictionary of gestures can force humans to communicate briefly. Therefore, gesture recognition is a good way to avoid some communication drawbacks, but in our rescue gestures, we need to choose the most representative gestures according to the different cultural backgrounds [20].

Human gesture recognition technology [21,22,23,24] is an emerging topic in drone applications. Compared to wearable sensor-based approaches [25,26], automated methods for video analysis based on computer vision technology are almost non-invasive. The control of drones by gesture recognition has already been implemented [27]. However, most of the datasets available in this field are still limited to indoor scenarios, and therefore, it is necessary to develop more and more outdoor UAV datasets. Many researchers are currently contributing to the lack of such outdoor drone datasets, for example, an outdoor recorded drone video dataset for action recognition [28], an outdoor dataset for UAV control and gesture recognition [29], and a dataset for object detection and tracking [30], among others. However until now, there is not suitable outdoor dataset to describe some of the generic gestures that humans make when they are in the wilderness environment. In this work, a data-set of ten body rescue gestures (i.e., Kick, Punch, Squat, Stand, Attention, Cancel, Walk, Sit, Direction, and PhoneCall) has been created by a UAV on-board camera. The number 10 is an approximate number based on some of the literature cited, which is in the range of effective communication. The two most important dynamic gestures are the novel dynamic Attention and Cancel which represent the set and reset functions respectively. We use this newly created dataset (detailed in Section 2.2) and the hand gesture dataset (detailed in Section 2.3) for human gesture recognition, combining from overall body to local hand gestures for better rescue results. The motivation for this paper is as follows: the first step is to find human bodies, and the second step is body gesture recognition in order to make human UAV interaction by Attention and Cancel gestures. People coming to the foreground and making “Attention” dynamic gesture is for the further investigation. The last step is further communication with recognizing hand only happens when the user shows Attention body gesture.

Communication between the user and the drone is achieved through the user’s body gesture recognition. Short and effective user feedback during this communication process can greatly improve the efficiency of the rescue. Based on the 10 basic body rescue gestures created in this work, we have chosen a pair of dynamic gestures: a two-handed waving motion (Attention) and a one-handed waving motion (Cancel) [31] as the two most basic communication vocabularies, well separated from the static gesture patterns. When the user extends both arms to call the drone, the drone will issue a warning and go into help mode. The system moves to the next stage where the drone slowly approaches the user in high resolution for localized hand gesture recognition. When a human extends only one arm, it means that the user has to cancel communication with the drone. In other words, the user does not need any help and the system is switched off. The dynamic gestures Attention and Cancel take on the functions of setting and resetting respectively in the system. For people who do not want to interact with the drone, (e.g., standing people), then no alarm will be issued. The cancellation gesture idea comes from a user-adaptive hand gesture recognition system with interactive training [31]. These dynamic gestures have been introduced in our paper [31], to avoid the problems with the impossible need for a pre-trained gesture-pattern database, since they allow the modification and restart. In paper [32], a temporal “freezing” was used for reinforcement/cancellation procedure. Following our earlier solution, try to make the untrained user to use our system easily based on general gesture languages in this paper.

Novelties and main issues of the methodology in the paper:

- A limited and well-oriented dictionary of gestures can force humans to communicate with UAV briefly during the rescue. So gesture recognition is a good way to avoid some communication drawbacks for UAV rescue.
- A dataset of ten basic body rescue gestures (i.e., Kick, Punch, Squat, Stand, Attention, Cancel, Walk, Sit, Direction, and PhoneCall) has been created by a UAV’s camera, which is used to describe some of the body gestures of humans in a wilderness environment.
- The two most important dynamic gestures are the novel dynamic Attention and Cancel which represent the set and reset functions respectively, well separated from the static gesture patterns.
- The combination of whole body gesture recognition at a distance and local hand gesture recognition at close range makes drone rescue more comprehensive and effective. At the same time, the creation and application of these datasets provide the basis for future research.

In the subsequent sections, Section 2 presents technical background and related work, including machine specifications, UAV connectivity, and gesture data collection strategies. In Section 3, the proposed methodology is presented, followed by human detection, pose extraction, human tracking and counting, body rescue gesture recognition, and proximity hand gesture recognition, along with a description of the relevant models and training and system information. Finally, Section 4 discusses the training results of the models and the experimental results. Conclusions and future work are drawn in Section 5.

## 2. Technical Background and Related Work

### 2.1. Machine Specification and UAV Connection

**Figure 1:**
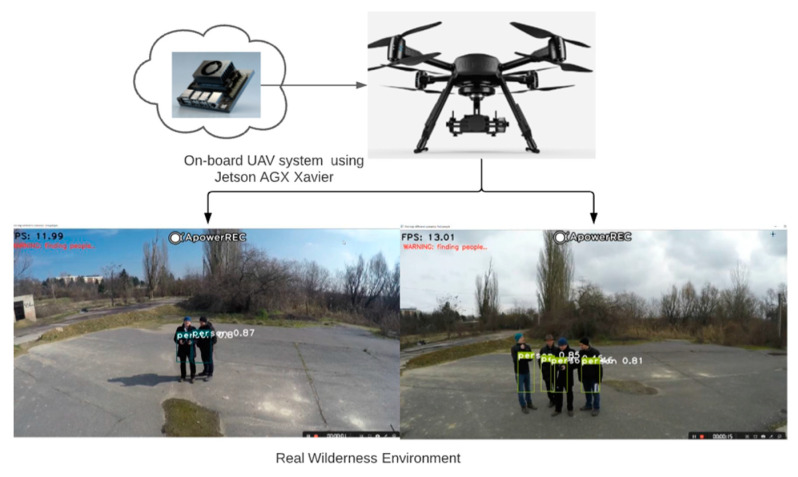
Practical application of an on-board unmanned aerial vehicles (UAV) with Jetson Xavier GPU for rescue.

**Figure 2:**
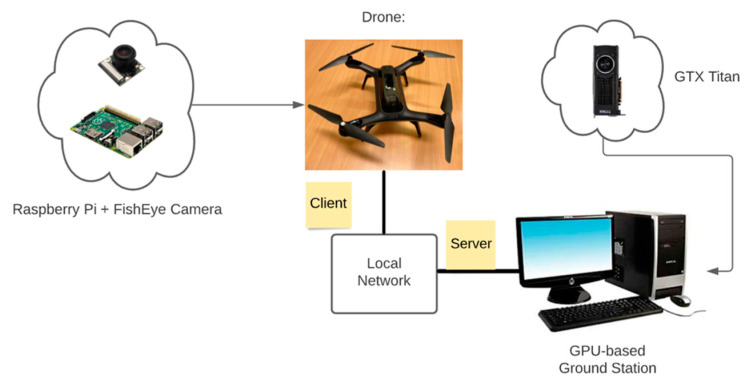
Training and local testing configuration of a Raspberry Pi system UAV with GPU-based ground station for rescue.

Figure 1 shows that the practical implementation of this work was done on an on-board UAV with Jetson Xavier GPU. Stand-alone on-board system is crucial, since in the wilderness we do not have network to rely on. From Sabir Hossain’s experiments [33] on different GPU systems, it was evident that Jetson AGX Xavier was powerful enough to work as a replacement of ground station for a GPU system. This is the reason why the Jetson Xavier GPU has been chosen. In the fourth chapter of this paper, in the experimental session, to ensure the reliable testing conditions we could not go to the field to fly the UAV for some external reasons, so we simulated the field environment in the lab and prepared for UAV motion control. The system for the testing part was changed, as shown in Figure 2. The testing part was done on a 3DR SOLO drone based on the Raspberry Pi system [34], which relies on a desktop ground station with GTX Titan GPU. The drone communicates with the computer through a local network. The comparison of the two GPUs is in Table 1 [35]. In Chapter 4 we also tested the real running time of the program on the proposed architecture.

For the testing part in the lab, the ground station computer is equipped with an NVIDIA GeForce GTX Titan GPU and an Intel(R) Core (TM) I7-5930k CPU, which is also used for model training. The UAV is a raspberry pi drone, which is a single-board computer with a camera module and a 64-bit quad-core ARMv8 CPU. The type of camera is a 1080P 5MP 160° fish eye surveillance camera module for Raspberry Pi with IR night vision. Table 2 presents the specification of the 3DR SOLO drone and the camera.

The resolution of the camera is to be changed according to the different steps of operation of the system. The resolution of the drone camera is set to 640 × 480 for human detection and body gesture recognition and 1280 × 960 for hand gesture recognition. In the test, the drone flies at an altitude of about 3 m in the laboratory with the camera resolution set as above. The higher the resolution of the drone’s camera, the higher the altitude at which the drone can fly, thinking of the minimal resolution needed for recognition. Therefore, the system can also work well at heights of more than ten meters with a high-resolution sensor of the UAV camera.

### 2.2. Body Gesture Data-Set Collection

**Figure 3:**
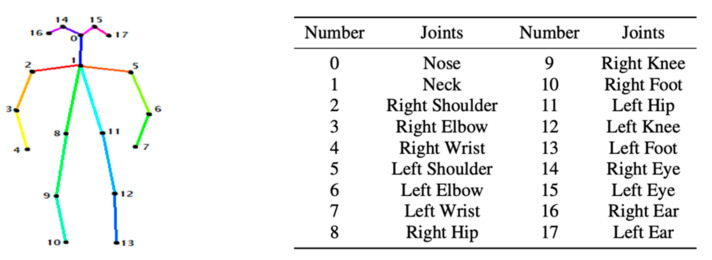
OpenPose Skeleton data and joints information.

OpenPose [36] is a real-time multi-person framework displayed by the Perceptual Computing Lab of Carnegie Mellon College (CMU) to identify a human body, hand, facial, and foot key points together on single images. Based on the robustness of the OpenPose algorithm and its flexibility in extracting key points, we use it to detect the human skeleton and obtain skeletal data in different gestures of the body, thus laying the data foundation for subsequent recognition. The key thought of OpenPose is employing a convolutional neural network to produce two heap-maps, one for predicting joint positions, and the other for partnering the joints into human skeletons. In brief, the input to OpenPose is an image and the output is the skeletons of all the people this algorithm detects. Each skeleton has 18 joints, counting head, neck, arms, and legs, as appeared in Figure 3. Each joint position is spoken to within the image arranged with coordinate values of x and y, so there’s an add up to 36 values of each skeleton. Figure 3 shows the skeleton data and key points information.

There are no publicly available and relevant datasets in the field of the wilderness rescue of humans by drones. To deal with this problem, based on our preliminary work [37], we create a new dataset dedicated to describing brief and meaningful body rescue gestures made by humans physically in different situations. Considering that people in different countries have different cultural backgrounds, some gestures may represent different meanings. Therefore, we have selected and defined ten representative rescue gestures that are used to convey the clear and concrete messages without ambiguity that humans make in different scenarios. These gestures include Kick, Punch, Squat, Stand, Attention, Cancel, Walk, Sit, Direction and PhoneCall. This dataset can of course be extended to a larger dataset.

In our dataset, the emphasis is on two dynamic gestures, Attention and Cancel, well separated from the static gesture patterns, as these represent the setup and reset functions of the system. The system will only alert when these two gestures are recognized. Attention represents the need for the user to establish communication with the drone, which will fly toward the user for further hand gesture recognition. Conversely, Cancel sends an alert that the user does not need to establish contact and the system is automatically switched off. The system will not sound an alarm when other rescue gestures are recognized. Except for Attention and Cancel, the remaining eight body gestures are considered as signs of normal human activity and therefore do not interact further with the drone. However, this is not absolute, for example, we can also set the PhoneCall gesture as an alarming gesture according to the actual demand, and when the user is recognized to be in the PhoneCall gesture, the drone quickly issues an alarm and later goes to recognize the phone number given by the user through the hand gesture, which can also achieve the rescue purposes. However, this specific function will not be discussed in this paper, because the latter hand gesture dataset we collected is limited and no recognition of numbers is added. From the gesture signs of usual human activity, we can build up a limited but effective clear vocabulary set for communicating simple semantics. It is not the task of the present paper, but it will be developed in the future; now the emphasis is on the gesture-based communication.

The datasets are collected using a 1080P 160° fish eye surveillance camera module for raspberry pi on the 3DR SOLO UAV system. Six people from our lab participated in UAV body rescue gesture data collection and real-time prediction, the genders were four males and two females, aged btw 22 and 32 years old. They also have performed each rescue gestures with all the possible variations. The proposed system recognizes the very common ten body rescue gestures in real-time, including the ones listed above. In these ten body gestures, we collected as many as possible of the two gestures of Attention and Cancel, to make the system’s setup and reset functions more powerful. It is important to note that this dataset describes the gesture signs of usual human activity that humans would make in a wilderness environment. Not all gestures will sound an alarm for rescue. Table 3 describes the details of each UAV body rescue gesture. Table 4 describes the details of the UAV body rescue dataset.

### 2.3. Hand Gesture Data-Set Collection

Based on the description in Section 2.2, when the user sends a distress signal to the drone, in other words, when the drone recognizes the human gesture as Attention, the system enters the final stage for hand gesture recognition and the drone automatically adjusts the resolution to 1280 × 960, while slowly approaching the user needs assistance. For hand gesture recognition, there are already many widely used datasets, the dataset for hand gesture recognition in this work is partly derived from GitHub [38] and partly defined by ourselves. We have adapted some of the gesture meanings to the needs. An outstretched palm means Help is needed, an OK gesture is made when the rescue is over, the gestures Peace and Punch are also invoked. Finally, we added Nothing for completeness of hand gesture recognition. In addition to the above four hand gestures, we also added the category Nothing, and in the dataset we collected some blank images, arm images, partial arm images, head images, and partial head images to represent the specific gesture of Nothing. Table 5 shows the details of hand gesture dataset.

We use hand gesture recognition to allow the drone to go further in discovering the needs of the user. Whole-body gesture recognition is distant, while hand gesture recognition is close. The combination of the whole body gesture and the partial hand gesture can make the rescue work more adequate and meaningful. We chose the limited gesture vocabularies for rescue gesture recognition to force the user to communicate briefly and effectively with the drone under certain conditions. Compared to speech recognition rescue, gesture recognition is a better way to get rid of the interference of the external environment.

Here we have selected only five hand gestures for recognition, but of course, we could also include more gestures such as numbers. As we discussed in Section 2.2, when the user’s body gesture recognition in the previous phase resulted in a PhoneCall, then the user can also give the phone number to the drone by using hand number gesture recognition. We refer here to the result of Chen [39], what will be developed in the future phase. From the gesture signs of usual human activity, we can build up a limited but effective clear vocabulary set for communicating simple semantics. In the hand gesture recognition phase, we can also capture the new hand gestures given by the user and later enter the names of the new gestures. By retraining the network, we can get the new model of gesture recognition with the new hand gesture of the user. Hand gesture prediction is shown in Chapter 4 by a real-time prediction bar chart.

## 3. Methodology

**Figure 4:**
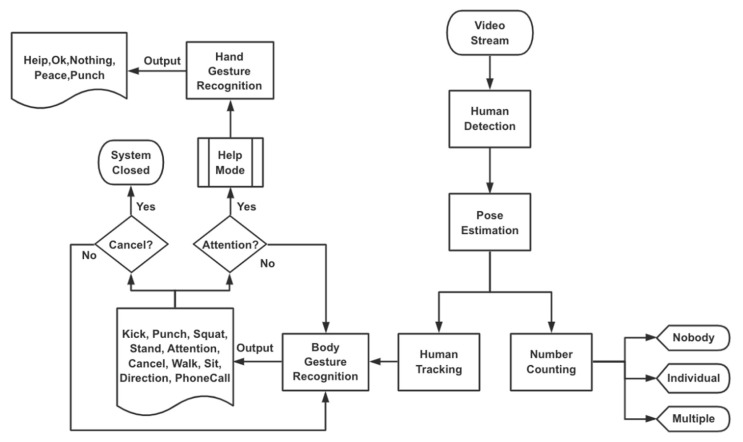
Framework of the whole system.

The system framework proposed in this paper is based on rescue gesture recognition for UAV and human communication. In this section, human detection, counting, and tracking are described. Body gesture recognition with set and reset functions and hand gesture recognition at close range are explained in detail. Figure 4 shows the framework of the whole system. First, the server on the onboard action unit drone side is switched on and the initial resolution of the drone camera is set. The input to the system is the live video captured by the drone‘s camera and the process is as follows: in the first step human detection is performed and when a person is detected by the drone, the system proceeds to the next step of rescue gesture recognition. In the second step, pose estimation is performed by OpenPose and the human is tracked and counted. The third step is the recognition of the body rescue gestures. Feedback from the human is crucial to the UAV rescue. The cancellation gesture idea comes from our user-adaptive hand gesture recognition system with interactive training [31]. When the user’s body gesture recognition results in Attention, the system proceeds to the final step of hand gesture recognition. If the user’s body gesture recognition is a cancellation, then the system switches off directly and automatically. The system uses gesture recognition technology to force the user to communicate briefly, quickly, and effectively with the drone in specific environments.

### 3.1. Human Detection

YOLO [40,41] is an open-source state-of-the-art object detection framework for real-time handling. Using a completely different approach, YOLO has a few advantages, compared to earlier region object detection systems and classification systems, within the way it performs detection and prediction. Region proposal classification systems perform detection by applying the model to an image with multiple predictions in different image regions and scales. High-scoring regions are considered as detections, however, YOLO uses a one-stage detector methodology and its design is similar to a fully convolutional neural network. The advantage of YOLO for real-time object detection is the improvement of deep learning-based location method. In our system, high speed is required. Previous YOLO versions apply a softmax work to convert scores into probabilities with an entirety rise to 1.0. Instead, YOLOv3 [42] uses multi-label classification by replacing the softmax function with free logistic classifiers to calculate the probability of an input belonging to a specific label. Hence, the model makes multiple predictions over different scales, with higher accuracy, in any case of the predicted object’s size.

Considering the real-time problem of our proposed system, this paper selects yolo3-tiny [42] for human detection. The dataset used in this method is a widely used COCO dataset [43], which contains a total of 80 categories of objects. Comprising a change of YOLO, yolo3-tiny treats detection to some degree differently by predicting boxes on two different scales whereas features are extracted from the base network. Its higher performance compared to YOLO was the most important reason for its selection. The model’s architecture consists of thirteen convolutional layers with an input size of 416 × 416 images. Although it can detect the 80 objects provided by the COCO dataset very well, in our system we only need to detect people. When the object category detected by the UAV is a person, the system will issue an alarm and then proceed to the next human gesture recognition. The main aim of the first stage is to find the human, if no human is detected then the system will remain in this stage until the drone detects a human.

### 3.2. Body Gesture Recognition

**Figure 5:**
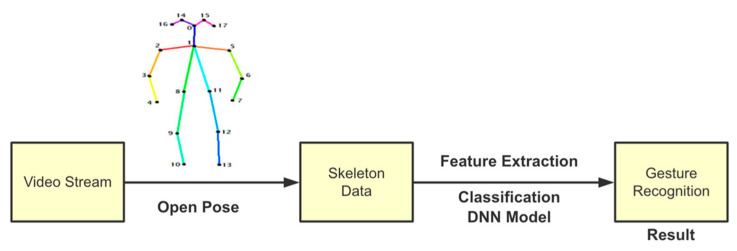
Workflow of the human body gesture recognition.

Figure 5 shows the flowchart for the human body gesture recognition. OpenPose algorithm is adopted to detect human skeleton from the video frames. These skeleton data are used for feature extraction, which is then fed into a classifier to obtain the final recognition result. We make the real-time pose estimation by OpenPose through a pre-trained model as the estimator [44]. OpenPose is followed by Deep Neural Network (DNN) model to predict the user’s rescue gesture. The Deep SORT algorithm [45] is used for human tracking for the multiple people scenario. The main reasons for choosing this latest method are as follows. Human tracking is not only based on distance and velocity but also based on the features that a person looks like. The main difference from the original SORT algorithm [46] is the integration of appearance information based on a deep appearance descriptor. Deep SORT algorithm allows us to add this feature by computing deep features for every bounding box and using the similarity between deep features to factor into the tracking logic.

After OpenPose skeleton extraction and Deep SORT human tracking, we can obtain information about human beings. By counting the number of people, we finally determined the following three scenarios: nobody, individual, and multiple people. If the drone does not detect anyone, then the communication between the drone and the user will not be established and the gesture recognition is fruitless. If the drone detects one or more people, then the drone will enter the gesture recognition phase for those people and show different recognition results based on the user’s body gesture to achieve communication between the user and the drone to assist humans. We are mainly concerned with the two gestures Attention and Cancel, which represent the two functions of setting and resetting respectively, so when these two gestures appear, the system will show a warning, turn on help mode or cancel the interaction.

Compared to other gesture recognition methods, such as using 3D convolutional neural networks [47], we finally chose the skeleton as the basic feature for human gesture recognition. The reason is that the features of the human skeleton are concise, intuitive, and easy to distinguish between different human gestures. In contrast, 3DCNN is both time-consuming and difficult to train large neural networks. As for the classifiers, we experimented with four different classifiers, including kNN [48], SVM [49], deep neural network [50], and random forest [51]. The implementation of these classifiers was from the Python library “sklearn.” After testing the different classifiers the DNN was finally chosen and the DNN showed us the best results.

The DNN model has been programmed using Keras Sequential API in Python. There are four layers with batch normalization behind each one and 128, 64, 16, 10 units in each dense layer sequentially. The last layer of the model is with Softmax activation and 10 outputs. The model is applied for the recognition of body rescue gestures. Based on the establishment of the above DNN model, the next step is training. The model is compiled using Keras with TensorFlow backend. The categorical cross-entropy loss function is utilized because of its suitability to measure the performance of the fully connected layer’s output with Softmax activation. Adam optimizer [52] with an initial learning rate of 0.0001 is utilized to control the learning rate. The demonstration has been trained for 100 epochs on a system with an Intel i7-5930K CPU and NVIDIA GeForce GTX TITAN X GPU. The total training dataset is split into two sets: 90% for training, and 10% for testing. Specific information such as the final body gesture recognition model accuracy and loss is described specifically in Section 4.

### 3.3. Hand Gesture Recognition

Further interaction with the drone is established by the user through an Attention body gesture. Whether it is a single person or a group of people, the drone enters help mode whenever a user is recognized by the drone in a body gesture of Attention. The camera resolution is automatically adjusted to 1280 × 960 as the drone slowly approaches the user. This is the final stage of this system, which is hand gesture recognition.

**Figure 6:**
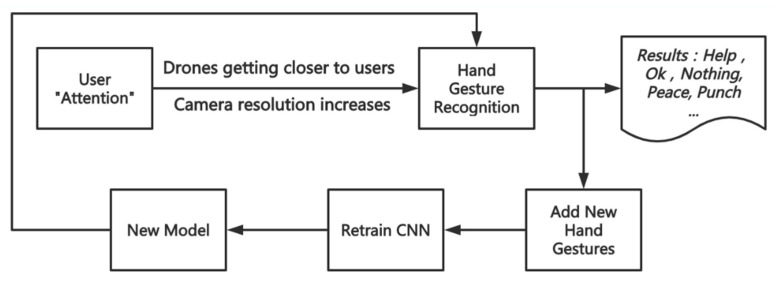
Workflow of the human hand gesture recognition.

Figure 6 shows the flowchart regarding this section. Hand gesture recognition is implemented by using a convolutional neural network (CNN) [53]. The 12-layer convolutional neural network model is compiled using Keras with TensorFlow backend. The CNN model can recognize 5 pre-trained gestures: Help, Ok, Nothing (i.e., when none of the above gestures are input), Peace, Punch. The system can guess the user’s gesture based on the pre-trained gestures. A histogram of real-time predictions can also be drawn. The combination of recognition of overall body gesture at a distance and hand gesture at a close distance makes drone rescue more comprehensive and effective. Although the gestures that can be recognized at this stage are limited, the system can also capture and define new gestures given by the user as needed and get a new model by retraining the CNN. As an example, we can add the recognition of numbers by human hand gestures as described before in Section 2.3, when the body gesture recognition in the previous section results in a PhoneCall, at which point the two can be used in combination, and the user can provide the drone with the phone number to be dialed via hand gesture recognition, thus also allowing for rescue purposes.

The dataset has a total of 4015 gesture images in 5 categories, with 803 image samples in each category. The total dataset is split into two sets: 80% for training, and 20% for testing. After training for 20 epochs, the model achieves 99.77% precision on training data and 94.71% accuracy on testing data.

## 4. Experiment

**Figure 7:**
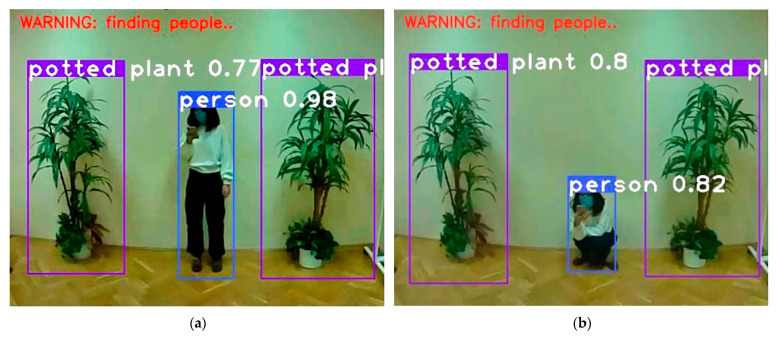
Human detection standing (**a**) and squatting (**b**) by using yolo3-tiny.

In this section, the model and performance of the proposed human detection and rescue gesture recognition system for UAVs are described as follows. Based on the introduction in Chapter 2, the testing phase of the designed system was done in the laboratory in a simulated field environment, and Table 6 shows the real running time required for each phase of the program to run on a proposed Jetson AGX Xavier GPU-based UAV. It should be noted that the results below are cutting images, and the original image should be in a 4 to 3 ratio, as we have tried to recreate the field environment without some clutter such as tables and chairs that we did not want to be included, so we have cut a fixed area of the output video. Figure 7 shows the results of human detection via yolo3-tiny. It is worth bringing up the point that we have simulated wild forest scenarios in the lab, but of course, it can detect humans in other scenarios as well. We can see that based on the COCO dataset, plants, squatting, and standing persons can be detected. If no person is detected, the system will not display a warning. Immediately after the warning appears the system goes into the recognition phase of the human rescue body gestures.

**Figure 8:**
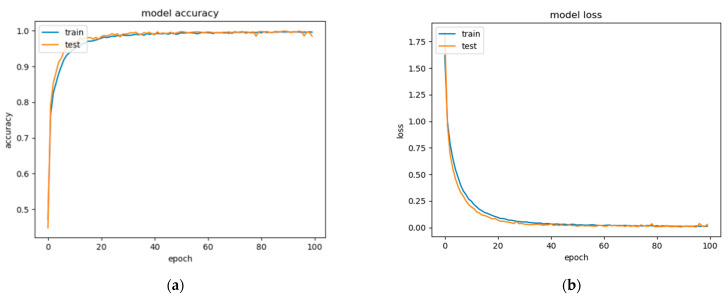
Body gesture recognition model accuracy (**a**) and loss (**b**) over the epochs.

Based on the body rescue gesture dataset created in Table 3, we trained the model through a deep neural network to finally obtain the accuracy and loss of the body gesture recognition model. The changes in accuracy and loss function are shown in Figure 8 over the course of training. At first, the training and testing accuracies increase quickly. Afterward, slow growth between 10 epochs and 20 epochs and merging happens after 25 epochs. The accuracy and loss approach to their asymptotic values were seen after 40 epochs with minor noise in between. The weights of the best fitting model with the highest test accuracy are preserved. Both, training as well as testing loss diminished consistently and converged showing a well-fitting model.

**Figure 9:**
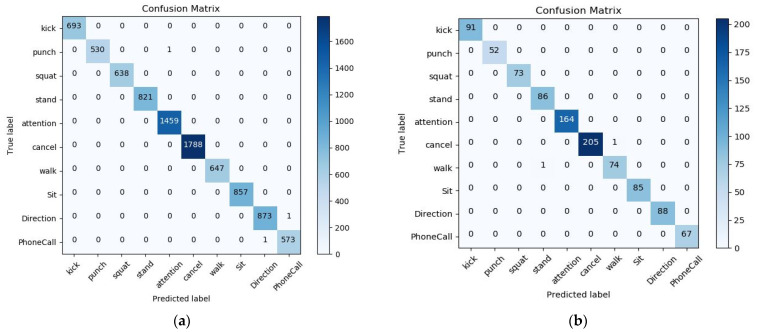
Confusion matrix with predicted labels on *X*-axis and true labels on the *Y*-axis tested on body gesture training set (**a**) and in testing dataset (**b**).

**Figure 10:**
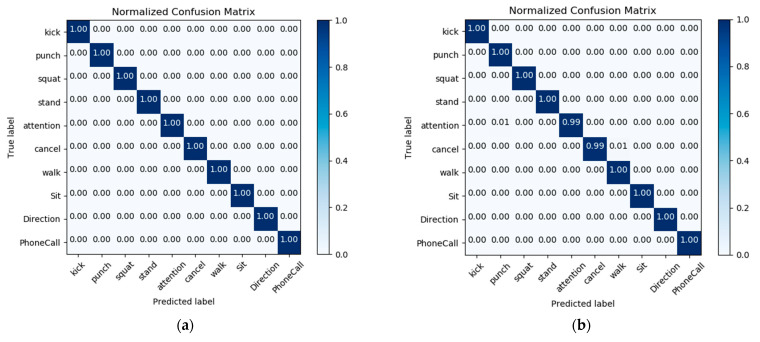
Standard matrix with predicted labels on *X*-axis and true labels on the *Y*-axis tested on body gesture training set (**a**) and in testing dataset (**b**).

After training for 100 epochs, the model achieves 99.79% precision on training data and 99.80% accuracy on testing data. In Figure 9, the diagram on the left presents the confusion matrix with predicted labels on *X*-axis and true labels on the *Y*-axis for predictions utilizing our model tested on the training dataset. The diagram on the right presents the confusion matrix with predicted labels on *X*-axis and true labels on the *Y*-axis for predictions utilizing our model on the testing dataset. The high density at the diagonal shows that most of the body rescue gestures were predicted correctly. The performance is well over and close to perfect in most of the gestures. In the confusion matrix, we can see that the amount of data for Attention and Cancel is relatively large. This is because, in the data collection part, we collect the largest amount of data for Attention and Cancel. These two gestures are dynamic body gestures and well separated from the static gesture patterns, which represent the set and reset functions respectively. In Figure 10, the diagram on the left presents the standard matrix with predicted labels on *X*-axis and true labels on the *Y*-axis for predictions utilizing our model tested on the training dataset. The diagram on the right presents the standard matrix with predicted labels on *X*-axis and true labels on the *Y*-axis for predictions utilizing our model on the testing dataset. The standard matrix is a scale for correctly identified gestures and mistakes, it shows that all body gestures in the training set have reached 1.00, and in the test set, all body gestures except Punch 0.98, Attention 0.99, and Walk 0.99 also reach 1.00. The sum of each row in a balance and normalized confusion matrix is 1.00, because each row sum represents 100% of the elements in a particular gesture. In addition to using the confusion matrix as an evaluation metric, we also analyzed the performance of the model from other standard metric. we use the equations below to calculate the macro-average. Based on the true positive (TP), false positive(FP), false negative(FN), and true negative(TN) of the samples, we calculate the *p* value (Precision), and R value (Recall), respectively, and the result macro F1 value is mostly close to 1.00.

$$\mathrm{Precision}\;=\frac{TP}{TP+FP}\;,\;\mathrm{Recall}\;=\frac{TP}{TP+FN}$$

$$\mathrm{macro}P=\frac{1}{n}\sum_{i=1}^{n}P_{i},\mathrm{macro}R=\frac{1}{n}\sum_{i=1}^{n}R_{i}$$

$$\mathrm{macro}F1=\frac{2\times \mathrm{macro}P\times \mathrm{macro}R}{\mathrm{macro}P+\mathrm{macro}R}$$

As communication between the drone and the GPU-based ground station in the lab is dependent on the local network, requests sent from the client-side and accepted by the server directly reduce the value of the FPS, causing the system to run very slowly. The system only reaches approximately 5 FPS in a real-time operation. But running directly on a drone loaded with a Jetson Xavier GPU would solve this problem, i.e., a practical application scenario, as shown in Figure 1. It has a Jetson Xavier GPU as powerful as the ground station (GTX Titan GPU) and does not need to communicate over the local network, it will be fast enough to meet practical needs. In the laboratory tests, the drone was always flown at an oblique position above the person, approximately 2 to 3 m away from the user in the hand-gesture recognition (close) position. The oblique position ensures that the entire human body can be recognized with a higher probability than flying directly above the user’s head and downwards vertically. Because the work is based on the human skeleton, the flying position of the drone has some limitations on the recognition results.

**Figure 11:**
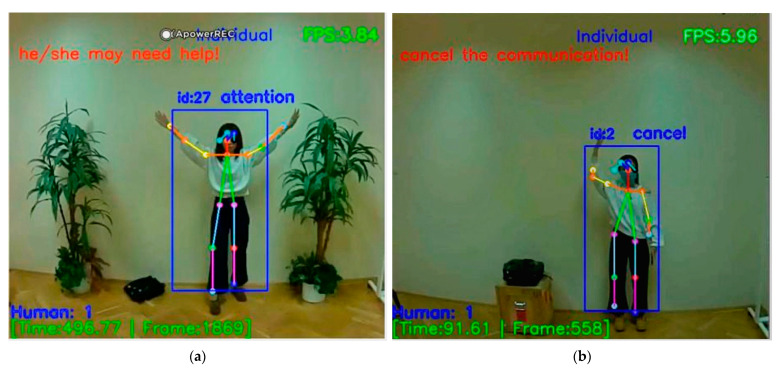
Attention gesture recognition (**a**) and Cancel gesture recognition (**b**).

**Figure 12:**
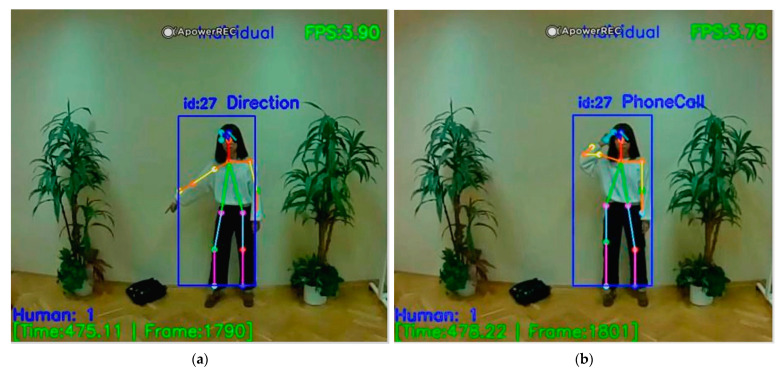
Direction gesture recognition (**a**) and PhoneCall gesture recognition (**b**).

Figure 11 shows the recognition of the Cancel gesture and Attention gesture with warning messages in real-time. Figure 11 also gives information about the number of people, time, frame, and FPS. Next are the recognition display and detailed description of two basic gestures that we randomly selected from the dataset. In Figure 12, the diagram on the left shows us that when a user points in a specific direction, the purpose is to alert the drone to look in the direction the person is pointing to. For example, when the direction pointed has someone lying on the ground, this gesture solves the problem that when somebody lying on the ground, UAV cannot recognize the skeleton information about the lying person well due to flight position of the drone. Direction gesture is also helpful to the fainted or unconscious people, when there is a group of people, those who have motion can use the Direction gesture to give instructions to the drone to save those who cannot move. Practically, as the main issue, our proposed system is for helping people in a bad situation, but we do not want to disturb persons who do not want or could not interact. The on-board system may send messages to the central about non-moving people, but we leave them in peace if they simply have a rest. In Figure 12, the diagram on the right shows the user’s gesture to make a phone call, which can be linked to hand gesture number recognition at a later stage. When the user poses to make a call, we can perform hand number recognition at a later stage to get the phone number the user wants to dial.

**Figure 13:**
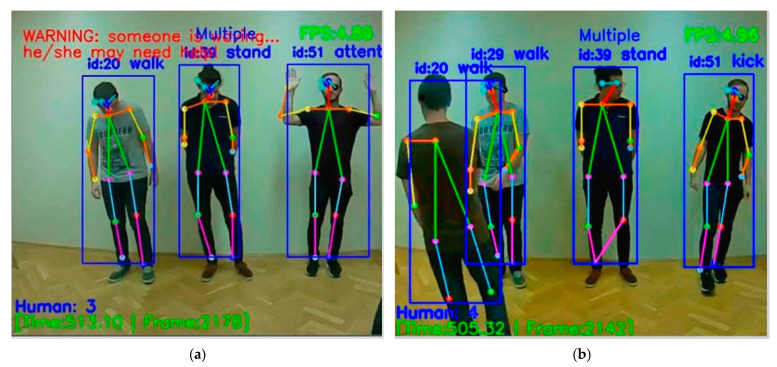
Multiple people with (**a**) and without (**b**) communication with UAV.

During the human body gesture recognition, Attention and Cancel are dynamic gestures that function as set and reset respectively and should therefore confuse the UAV board recognition during the frame-by-frame check. When either of these two gestures is detected, the system will immediately give an alert. Figure 13 shows that when there are multiple people, one of them sends an Attention gesture to the drone. At this point, the drone sends a warning to inform that someone needs help. We can also see in Figure 12 that other people’s gestures are well recognized in addition to the person making the Attention gesture. In our recognition system, about 10 people can be recognized at the same time during human body gesture recognition. Figure 13 also shows the basic gesture recognition of multiple people without warning. We can see some people standing, some people walking, and some people kicking. Also, the number of people, time, frame, and FPS will be displayed. It should be noted that if a person is not fully present in the drone camera, then it will not be recognized. People’s movements are generated continuously in real-time, and Figure 13 is a photo we took from the video, so there will be some inaccurate skeleton information. Of course, if a person’s gesture is not in our dataset, that person’s gesture will not be recognized and the recognition result information above it will be blank.

**Figure 14:**
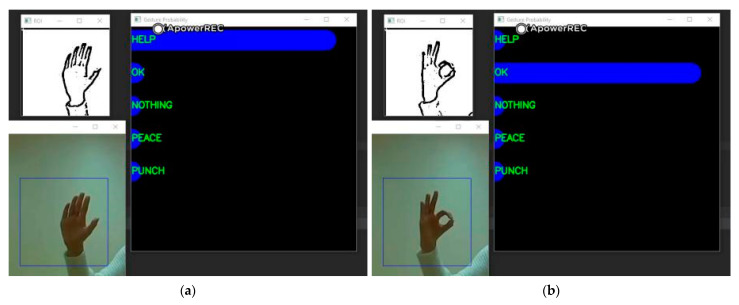
Help hand gesture recognition (**a**) and Ok hand gesture recognition (**b**).

**Figure 15:**
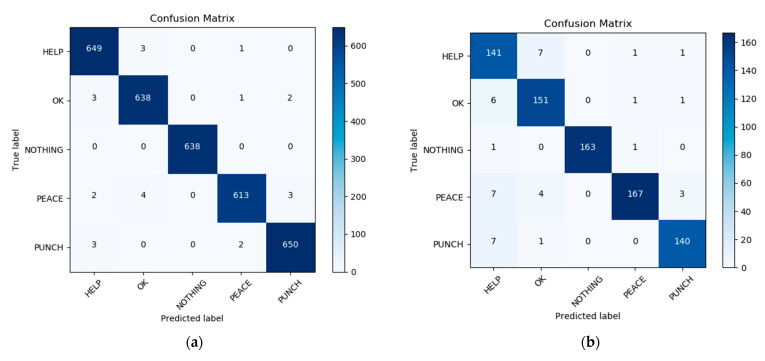
Confusion matrix with predicted labels on *X*-axis and true labels on the *Y*-axis tested on hand gesture training set (**a**) and in testing dataset (**b**).

**Figure 16:**
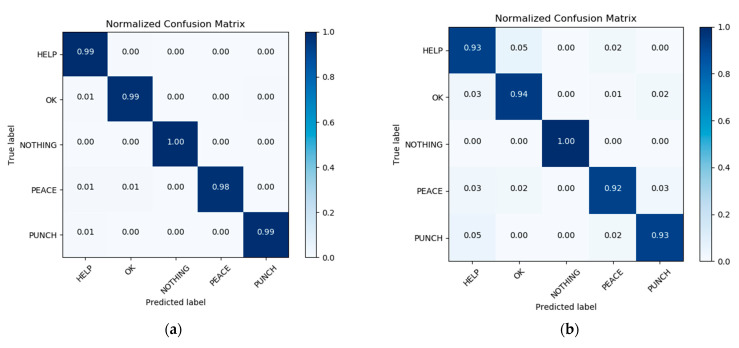
Standard matrix with predicted labels on *X*-axis and true labels on the *Y*-axis tested on hand gesture training set (**a**) and in testing dataset (**b**).

When the result given by the user in the previous stage is the body gesture of Attention, then the drone adjusts the resolution to 1280 × 960 and slowly approaches the user to perform the recognition of the hand gesture. We selected two more representative hand gesture recognition results to show, a Help gesture and an Ok gesture, where the user establishes further communication with the drone through the Attention body gesture in the previous stage. In the last close hand gesture recognition stage, the user can inform the drone that it needs to help him/her through the Help hand gesture, and when the drone is done helping the user, the user can inform it through the Ok hand gesture. Figure 14 shows us the results of the recognition of the Help and Ok gestures. From the displayed results we can see that the user’s hand gesture recognition results can be well predicted by the histogram. Of course, we can also capture and define new gestures for the user on a case-by-case basis and add the new gestures to the gesture dataset by retraining the network. In Figure 15, the diagram on the left presents the confusion matrix with predicted labels on *X*-axis and true labels on the *Y*-axis for predictions utilizing our model tested on the training dataset. The diagram on the right presents the confusion matrix with predicted labels on *X*-axis and true labels on the *Y*-axis for predictions utilizing our model on the testing dataset. The high density at the diagonal shows that most of the body rescue gestures were predicted correctly. The performance is well over and close to perfect in most of the gestures. In Figure 16, the diagram on the left presents the standard matrix with predicted labels on *X*-axis and true labels on the *Y*-axis for predictions utilizing our model tested on the training dataset. The diagram on the right presents the standard matrix with predicted labels on *X*-axis and true labels on the *Y*-axis for predictions utilizing our model on the testing dataset. The standard matrix shows that the corresponding values for the five categories of hand gestures can reach 0.99 or 1.0 on the training set and 0.9 or more on the test set.

## 5. Conclusions and Future Work

In this paper, we propose a real-time human detection and gesture recognition system for onboard UAV rescue. Practical application and laboratory testing are two different systems. The system not only detects people, tracks them, and counts the number of people, but also recognizes human rescue gestures in a dynamic system. First of all, the drone detects the human at a longer distance with a resolution of 640 × 480, and the system issues an alarm to enter the recognition stage when a person is detected. A dataset of ten basic body rescue gestures (i.e., Kick, Punch, Squat, Stand, Attention, Cancel, Walk, Sit, Direction, and PhoneCall) has been created by a UAV’s camera. The two most important dynamic gestures are the novel dynamic Attention and Cancel which represent the set and reset functions respectively, through which users can generate communication with the drone. After the Cancel gesture is recognized, the system automatically shuts down, and after the Attention gesture is recognized, the user can establish further communication with the drone. People coming to the foreground and making “Attention” dynamic gesture is for further investigation. The system enters the final hand gesture recognition stage to assist the user. At this point, the drone will automatically adjust the resolution to 1280 × 960 and gradually approach the user for close hand gesture recognition. From a drone rescue perspective, we did a good job of getting feedback from users. This work lays some groundwork for subsequent user rescue route design.

The detection of the human body is achieved through yolo3-tiny. A rescue dataset of 10 gestures is collected by using a fisheye surveillance camera for 6 different individuals in our lab. OpenPose algorithm is used to capture the user’s skeleton and detect their joints. We built a deep neural network (DNN) to train and test the model. After training for 100 epochs, the framework achieves 99.79% precision on training data and 99.80% accuracy on testing data. For the final stage of hand gesture recognition, we use data collected online combined with our definitions to obtain a relevant dataset, which is trained by a convolutional neural network to obtain a model to achieve hand gesture recognition. Gestures can also be added or removed as required. The drone flies at an altitude of approximately 3 m and is flown diagonally above the user, rather than directly above the user. However, there are some difficulties and limitations when the system applies to the real wildness. In practice, the proposed system is subject to some extreme weather conditions and resolution issues. Another limitation is the flying position of the UAV. The system proposed in this paper requires drones fly over people at an angle in order to detect the human body gestures more accurately, rather than in a vertical user overhead position. For gathering enough experiment data we need more time and battery life-time limits the real-life data-gathering. For this reason, real-life data are only used for demonstration in Figure 1, while the exhaustive testing needed laboratory-based environment.

The main innovations and contributions of this paper are as follows: First, it is worth affirming that gesture recognition for wilderness rescue can avoid the interference of the external environment, which is the biggest advantage compared to voice recognition for rescue. A limited and well-oriented dictionary of gestures can force humans to communicate briefly. So gesture recognition is a good way to avoid some communication drawbacks. Second, a dataset of ten basic body rescue gestures (i.e., Kick, Punch, Squat, Stand, Attention, Cancel, Walk, Sit, Direction, and PhoneCall) has been created by a UAV’s camera, which is used to describe some of the body gestures of humans in the wild. For the gesture recognition dataset, not only the whole body gestures but also the local hand gestures were combined to make the recognition more comprehensive. Finally, the two most important dynamic gestures are the novel dynamic Attention and Cancel which represent the set and reset functions respectively. It should confuse the UAV-board recognition when checking frame-by-frame with a system warning. The system switches to a warning help mode when the user shows Attention to the UAV, and the user can also cancel the communication with the UAV at any time as needed.

In future work, more generic rescue gestures and larger hand gesture data sets could be included. The framework can be executed in real-time recognition with self-training. The system can automatically retrain the model based on the new data in a very short time to get a new model with new rescue gestures. Last but not the least, we also needed to conduct outdoor tests on a drone carrying a Jetson Xavier GPU.

The interpretation of the gesture based communication without predetermined vocabulary and unknown users will be a great challenge to linguistic research. Attention and Cancellation dynamic gestures will have a main role in generating a dynamic linguistic communication.

## Figures and Tables

**Table 1:** Comparison of NVIDIA GeForce GTX TITAN and Jetson AGX Xavier.

	NVIDIA GeForce GTX TITAN	Jetson AGX Xavier
Pipeline	2688	512
CUDA cores	2688	512
Core clock speed/MHz	830	854
Boost clock/MHz	876	1137
Transistor count	7080 million	9000 million
Power consumption(TDP)/Watt	250	30

**Table 2:** Specification of the UAV and camera for testing.

System	Patch Size	Output Size
Fish eye surveillance camera	Still picture resolution	2592 × 1944
	Viewing angle	160 degrees
	Video	supports 1080p@30fps,720p@60fps
	Size	approx.23 × 22 mm/0.90 × 0.86 inch
Raspberry Pi 3 [27]	CPU	1.2 GHz 64-bit quad-core ARMv8 CPU
	GPU	Broadcom video core 4
	Memory	1G
	Storage	Support MicroSD
UAV-3DR SOLO	Height:	10 in. (25 cm)
	Motor-to-motor dimension:	18 in. (26 cm)
	Payload	1.1 lbs. (500 g)
	Range	0.5 miles ** (0.8 km)
	Maximum altitude	328 ft. (100 m)
	Estimated flight time	25 min *

** Range varies with location, antenna orientation, background noise and multi-path. * Flight time varied with payload, wind conditions, elevation, temperature, humidity, flying style, and pilot skill. Listed flight time applies to elevations less than 2000 ft above sea level.

**Table 3:** UAV body rescue gestures and corresponding key points.

Number	Name	Body Rescue Gesture	Number	Name	Body Rescue Gesture
1	Kick	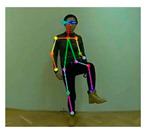	6	Cancel (Dynamic)	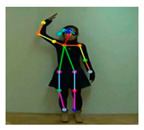
2	Punch	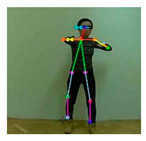	7	Walk	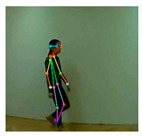
3	Squat	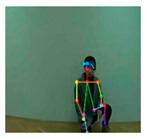	8	Sit	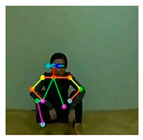
4	Stand	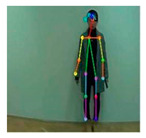	9	Direction	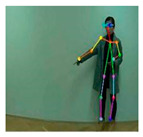
5	Attention (Dynamic)	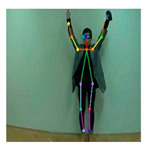	10	PhoneCall	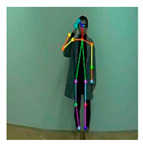

**Table 4:** UAV body rescue gestures dataset details.

Number	Name	Number of Skeleton Data
1	Kick	784
2	Punch	583
3	Squat	711
4	Stand	907
5	Attention	1623
6	Cancel	1994
7	Walk	722
8	Sit	942
9	Direction	962
10	PhoneCall	641

**Table 5:** UAV hand rescue gesture dataset.

Number	Name	Hand Rescue Gesture	Number of Images
1	Help	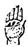	803
2	Ok	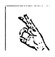	803
3	Nothing (something else)	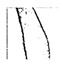	803
4	Peace	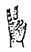	803
5	Punch	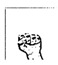	803

**Table 6:** Real running time of the proposed Jetson AGX Xavier GPU-based UAV platform.

Phase	Real Running Time
Human Detection	10 ms
Body Gesture Recognition	25 ms
Hand Gesture Recognition	20 ms

## Data Availability

The data presented in this study are available on request from the author.
